# Complete genome sequences of three co-occurring *Pseudomonas* isolates from California Botanic Garden topsoil

**DOI:** 10.1128/mra.00793-26

**Published:** 2026-09-04

**Authors:** Chun-Yu Chen, Xiaokuan Wang, Yan Yan, Rafael Escobar, J. Cesar Ignacio-Espinoza

**Affiliations:** 1 Kravis Department of Integrated Sciences, Claremont McKenna College 2530, Claremont, California, USA; 2 Riggs School of Life Sciences, Keck Graduate Institute 48927, Claremont, California, USA; 3 Harvey Mudd College 31750, Claremont, California, USA; Michigan State University, East Lansing, Michigan, USA

**Keywords:** *Pseudomonas*, soil

## Abstract

We report the complete genome sequences of three *Pseudomonas* isolates recovered from topsoil at the California Botanic Garden. Two isolates share ~99.4% average nucleotide identity, enabling investigation of intraspecies microvariation; the third represents a co-occurring distinct species, together capturing species- and strain-level genomic diversity within a natural soil community.

## ANNOUNCEMENT

High-fidelity, complete genomes of closely related environmental isolates, such as those of the abundant and diverse *Pseudomonas* genus (1), provide a valuable resource for studying microdiversity at full genome resolution. Here, we report the complete genome sequences of three *Pseudomonas* isolates recovered from soil samples at the California Botanic Garden (Claremont, CA).

Soil samples were collected using a T-Handle Soil Sampler Probe Rod Tool (Lichtund); see Table 1 for details of collection sites. Soil was stored in Ziploc bags, transported to Keck Graduate Institute, and stored at 4°C until further processing. Bacterial enrichments were performed using 5 g of soil in 25 mL of *Pseudomonas* Minimal Media (2 mM MgSO_4_, 0.31 mM FeCl_3_, 0.03 mM CaCl_2_, 15 mM NaH_2_PO_4_, and 15 mM K_2_HPO_4_, with pH adjusted to 6.8) amended with five drops of both Na-succinate (10% solution) and NH_4_Cl (20% solution) as carbon and nitrogen sources (2). After enrichment for 2 days, we plated the enrichments using a 1 μL inoculation loop on *Pseudomonas* minimal agar (1.5% agar). After 2 days of growth, colonies were picked and plated on *Pseudomonas* isolation agar (PIA; criterion no.: C6681; 500 mL milliQ water, 12.5 g PIA base medium, 10 mL glycerol). Single colonies were picked and grown overnight in lysogeny broth (LB; 25 g/L, 2.5% wt/vol) medium. The next morning, cultures with an optical density (OD600) of >0.6 were selected for storage. Selected cultures (650 μL) were taken and mixed with 50% glycerol solution in a 1:1 ratio, and these prepared samples were stored as stocks at −80°C. Multiple bacterial isolates were obtained during this process; however, the present study focuses specifically on three selected *Pseudomonas* isolates, for which we report complete genome sequences.

**TABLE 1:** Genome assembly, annotation, and collection metadata for the three *Pseudomonas* isolates^*a*^

Characteristic	Isolate 8-5-2	Isolate 9-2-1	Isolate 8-10-2
Sequencing sample ID	B9TF7H_1_Sample_1_8A	XH254M_1_sample_1	CZJZZ4_1_rerun_Sample_2_8B_2
Accession number	JCAFHX000000000	JCAFHY000000000	JCAFHW000000000.1
Raw read accession number (SRA)	SRR39379362	SRR39379361	SRR39379360
Isolation source	California Botanic Garden soil	California Botanic Garden soil	California Botanic Garden soil
Coordinates	34°6′54″ N, 117°43′28″ W	34°6′54″ N, 117°43′28″ W	34°6'54″ N, 117°43'28″ W
Elevation (m)	410	410	410
Adjacent plant	*Chilopsis linearis*	*Aesculus californica*	*Chilopsis linearis*
Sequencing platform	Oxford Nanopore (long read)	Oxford Nanopore (long read)	Oxford Nanopore (long read)
Genome size (bp)	5,929,752	6,178,779	5,698,233
Contigs (n)	1	1	1
Topology	Circular	Circular	Circular
N50 (bp)	5,929,752	6,178,779	5,698,233
GC content (%)	62.6	62.4	62.6
Sequencing depth (×)	95	94	96
Total reads	559,553	354,501	546,337
Read N50 (bp)	8,803	9,050	9,663
Median read length (bp)	3,474	3,436	3,839
Median read quality (Phred)	24.52	25.79	23.48
Completeness % (CheckM)	99.74	99.74	100.00
Contamination % (CheckM)	0.87	1.94	0.20
Closest species (GTDB)	*Pseudomonas_E sp013179515*	*Pseudomonas_E sp013179515*	*Pseudomonas_E capeferrum*
ANI to closest species (%)	99.75	99.53	98.66
Total genes	5,410	5,588	5,199
CDS (protein-coding genes)	5,264	5,449	5,055
tRNA	73	74	79
rRNA	22	22	22
ncRNA	50	42	42
Pseudogenes	10	11	12
Hypothetical proteins	177	233	172
Coding density (%)	89.7	89.6	90.9

^*a*^ Isolates are columns; metrics are rows. Coordinates are in degrees-minutes-seconds (WGS84); elevation is in meters. Completeness/contamination was determined by CheckM; the closest species and ANI were obtained by sourmash (GTDB).

For genome sequencing, colonies were grown from stocks on LB agar (1% agar) and then grown in 10 mL of LB media (2.5% LB). Overnight cultures were then pelleted at 3,000 g for 15 min and washed twice with PBS. DNA was extracted using the QuickDNA Kit (Zymo Research Corporation), following the manufacturer’s instructions. DNA was quantified using the Portlite Fluorimetric DNA Quantification Kit (AAT Bioquest) and renormalized to 50 ng/μL. Twenty microliters was sent out for sequencing at Plasmidsaurus (Eugene, OR) for Nanopore Sequencing using the ONT Ligation Sequencing Kit v14 (SQK0LSK1114) library preparation without DNA shearing and size selection on an R10.4.1 flow cell.

Default parameters were used for all software described in this study unless otherwise specified. Basecalling was performed with Dorado using the super-accuracy (sup) basecalling model (v4.3.0 [3]). Reads were quality-filtered with Filtlong (v0.2.1 [4]) using the flags “--min_length 300—keep_percent 95.” A *de novo* assembly was generated with Autocycler (v0.2.1 [5]) from input assemblies produced by Flye (v2.9.6 [6]), hifiasm (7) in ONT mode, and Plassembler (v1.8.0 [8]). The circular assembly graph was reoriented using dnaapler (v1.2.0 [9]). Genome completeness and contamination were assessed with CheckM (v1.2.2 [10]). Isolates were taxonomically classified with sourmash (v4.9.4 [11]) against a GTDB database, which assigned all isolates to the genus *Pseudomonas*, two with an average nucleotide identity (ANI) of 99.5%–99.75% to *Pseudomonas* sp013179515 (GCF_013179515.1) and one to *Pseudomonas capeferrum* (GCF_000731675.1, ANI 98.66%). Genomes were annotated with Bakta (v1.12.0 [12]). Raw sequencing data and general genomic features are summarized in Table 1.

## Data Availability

The complete genome of our isolates and raw sequencing data are available at PRJNA1482863 and PRJNA1483221, respectively. Individual accession numbers are listed in Table 1.
